# Electrostatic forces govern the binding mechanism of intrinsically disordered histone chaperones

**DOI:** 10.1371/journal.pone.0178405

**Published:** 2017-05-26

**Authors:** Chuanbo Liu, Tianshu Wang, Yawen Bai, Jin Wang

**Affiliations:** 1 State Key Laboratory of Electroanalytical Chemistry, Changchun Institute of Applied Chemistry, Chinese Academy of Sciences, Changchun, Jilin, P.R. China, 130022; 2 University of Chinese Academy of Sciences, Beijing, P.R. China, 130022; 3 College of Physics, Jilin University, Chuangchun, Jilin, P. R. China, 130012; 4 Laboratory of Biochemistry and Molecular Biology, Center for Cancer Research, National Cancer Institute, National Institutes of Health, Bethesda, Maryland, United States of America, 20892; 5 Department of Chemistry and Physics, State University of New York, Stony Brook, New York, United States of America, 11794-3400; Islamic Azad University Mashhad Branch, ISLAMIC REPUBLIC OF IRAN

## Abstract

A unified picture to understand the protein recognition and function must include the native binding complex structure ensembles and the underlying binding mechanisms involved in specific biological processes. However, quantifications of both binding complex structures and dynamical mechanisms are still challenging for IDP. In this study, we have investigated the underlying molecular mechanism of the chaperone Chz1 and histone H2A.Z-H2B association by equilibrium and kinetic stopped-flow fluorescence spectroscopy. The dependence of free energy and kinetic rate constant on electrolyte mean activity coefficient and urea concentration are uncovered. Our results indicate a previous unseen binding kinetic intermediate. An initial conformation selection step of Chz1 is also revealed before the formation of this intermediate state. Based on these observations, a mixed mechanism of three steps including both conformation selection and induced fit is proposed. By combination of the ion- and denaturant-induced experiments, we demonstrate that electrostatic forces play a dominant role in the recognition of bipolar charged intrinsically disordered protein Chz1 to its preferred partner H2A.Z-H2B. Both the intra-chain and inter-chain electrostatic interactions have direct impacts on the native collapsed structure and binding mechanism.

## Introduction

The ultimate goal of protein science is to uncover the relationship between protein sequence and function. Early efforts starting with the clues that protein structure is determined by its sequence and having stable 3-D structure seems to be the prerequisite for its function. These observations lead to the classical structure-function paradigm [1]. However, with the realization that some proteins, termed intrinsically disordered proteins (IDPs), can function *in vivo* without having stabilized structures, researchers are motivated to revisit the long holding idea that protein function is structurally determined [2]. Accumulating evidences now suggest a unified picture that includes both the folded and disordered proteins must consider the native structure ensembles as well as the binding mechanisms involved in specific biological function [3]. In other words, the sequence specific individual folding energy landscape and the global complex binding energy landscape should be directly related to protein functions together [4]. Recently, several enlightened functional features are proposed for the discussion of sequence-structure relationship of IDPs, namely, the linear motifs (LMs) [5], molecular recognition features (MoRFs) [6] and intrinsically disordered domains (IDDs) [7]. These structural features establish the linkage between functionality of proteins to disordered regions. On the other hand, the disordered region-mediated protein interactions lead to a combination of conformational selection and induced folding [8]. The dominant mechanisms are suggested to be dependent on the concentrations of individual proteins [9] and the association rate constants [10]. However, the whole picture is still controversial. Therefore, it is vital to understand how the disordered structural features influence the protein recognition mechanism and hence the way that proteins realize their functions.

For eukaryotic cells, the molecular machinery involved in packing the DNA chains into nucleosomes controls the cellular gene regulation processes such as replication, transcription, gene reparation and recombination of the DNA base pair [11, 12]. Among these regulation processes, histone chaperons are known to be the key players at all cellular regulation processes with the participations of histones and modulation of the dynamics for nucleosome during chromatin remodeling [13]. As a matter of fact, histone chaperones are interconnected with histone variants at the function level. The primary functions of eukaryotic histone chaperones are to shield the highly basic histones from aggregating by constructing heterodimer complexes and forming non-specific interactions with DNA [14]. The binding of histone chaperones also incorporates and exchanges canonical nucleosome histones with histone variants. The resulted variants therefore become the predominant species in the differentiated cell, modulate nucleosome structure and thus regulate chromatin dynamics [15]. The function of specific histone chaperone is likely conserved in all eukaryotic cells, since the histones and mostly all histone chaperones are sequentially conserved throughout evolution [16].

One newly discovered histone chaperon Chz1, which is characterized as an intrinsically disordered protein in physiological condition, has preference for the histone variant H2A.Z and functions by delivering the heterodimer H2A.Z-H2B for SWR1 complex which catalyzes the replacement of the canonical nucleosome H2A-H2B with H2A.Z-H2B in an ATP-dependent manner [17–19]. Interestingly, another chaperone Nap1 can also provide this source to the SWR1 chromatin remodeling machinery and is considered to have function overlap with Chz1 [20]. Native Chz1 is preferentially associated with histone variant H2A.Z over histone H2A, whereas Nap1 can ambiguously recognize (H3-H4)_2_ tetramers, H2A-H2B dimers as well as H2A.Z-H2B dimers and have non-specific interactions with DNA chains [21]. This specificity increase of chaperones Chz1 as compared with Nap1 is probably due to the flexibility of Chz1 intrinsically disordered native state, which can achieve a high specificity with moderate affinity [22]. In contrast, Nap1 is a stable obligate homo-dimer or self-associated oligomer in both solution and in the crystal [23]. Recent discovery has found Chz1 is not associated with H2A.Z in the cytoplasm while Nap1 is necessary for maintaining solubility of H2A.Z [24]. The deletion of Chz1 results in a significant decrease of H2A.Z binding at promoters and telomeres [25]. Nevertheless, Nap1 deletion dramatically increased the association of H2A.Z with chromatin and is dominant in gene expression regulation. With the observation that Chz1 does not bind to genomic DNA, the function of Chz1 may be realized by capturing the released H2A.Z-H2B dimers during nucleosome disassembly and delivering it to SWR1 for deposition [26]. The distinct functional role of Chz1 and Nap1 played in depositing histone variant H2A.Z on chromatin reflects the difference of molecular assemble mechanism of chaperones binding to H2A.Z. The histone chaperone activity of Nap1 can be attributed to its ability of lowering the free energy of the histone-chaperone complex by establishing contacts between chaperone and histone variant which is thermodynamically favored over non-specific histone variant-DNA interactions [27]. On the other hand, the binding of intrinsically disordered chaperone Chz1 blocks the histone hydrophobic surfaces, therefore increases histone solubility [28].

The DNA double-helix chains are highly negatively charged while histone proteins are extensively positively charged. Therefore, the electrostatic interactions play a crucial role in stabilizing the octamer structure, determining the nucleosome unwrapping pathway and modulating the interactions of histone proteins as well as facilitating the binding to their favorable partners, such as histone chaperones and gene regulators [29, 30]. In addition, intrinsically disordered histone chaperones have increased diffusive searching radius as compared with folded proteins [31]. These two factors are the essential parts of the fly-casting mechanism which speeds up molecular recognition by reducing the search time of protein monomers [31, 32]. Nevertheless, most IDPs are polyampholytes. In the case of opposite bipolar distribution sequences, the intra-chain long range electrostatic attractions will promote the collapse of the protein. This results in hairpin-like conformations in IDPs’ native states. The binding of Chz1 to H2A.Z-H2B is mediated with its middle region (residues 71-132) [17]. As an intrinsically disordered protein itself, Chz1 forms a long irregular chain caps by two short *α*-helices and makes contact with broad regions of H2A.Z-H2B with its middle region (71-132) while the conformation of TBZ is essentially the same as the free state, with the only minor conformation shifts being observed in the NMR relaxation experiments [33]. This intrinsically disordered region (IDR) can also be viewed as the molecular recognition features (MoRFs) of Chz1 since it undergo disorder to order transition when binding to H2A.Z-H2B. In the theoretical simulated transition states, the negatively charged N-terminal acidic motif of Chz1 interacts with the positively charged region of both H2B (K89, K90) and H2A.Z (R55, R57, K61). Meanwhile, the positively charged C-terminal basic motif of Chz1 has little inter-chain interactions [34]. These observations reveal the binding of Chz1 to H2A.Z-H2B had strong dependence on electrostatic interactions. Our previous simulation results had shown that Chz1 in low salt concentrations formed a tertiary collapsed structure due to the oppositely charged N- and C-terminal regions [34]. Also since the N-terminal region of Chz1 is largely negatively charged, the N-terminal binding pathway was suggested to be dominant in the recognition of Chz1 to H2A.Z-H2B. Therefore, the inter-chain electrostatic forces facilitate the formation of association complex and intra-chain electrostatic forces modulate the native structure ensembles of Chz1.

The dynamics and mechanisms of protein recognitions and interactions had been extensively studied with various physical experimental methods for understanding the forces and conformation changes in protein association [35–37]. Besides, genetic modification methods such as site-directed mutagenesis and amino acids modifications were also proved to be effective methods for study protein interactions [38]. Moreover, molecular modeling was essential in some cases for understanding the detailed molecular mechanism of protein folding and association [39]. In this study, to better understand the binding/folding process of Chz1 to H2A.Z-H2B, we performed both equilibrium and kinetic investigations to explore the underlying molecular mechanism of the Chz1-H2A.Z-H2B (CZB) complex association. The CZB complex had been found to dissociate easily with the increase of ionic strength, indicating that electrostatic interactions are vital for stabilizing the complex [40]. Therefore ionic strengths and urea concentrations were used in our experiments for perturbations. The equilibrium fluorescence and CD spectra were recorded with various ionic strengths and urea concentrations, and were fitted to an equilibrium two-state model. Furthermore, stopped-flow kinetic experiments were also performed with the solvent conditions varied. A numerical global fitting method was used in this study to extract the kinetic parameters. Free energy as well as kinetic rate constants dependence on electrolyte mean activity coefficients and urea concentrations were obtained. A hidden kinetic intermediate was revealed by the corresponding kinetic analysis and it is considered to be the encounter complex of Chz1 binding to H2A.Z-H2B. An initial conformation selection of Chz1 may appear before the formation of the intermediate state and a mixed mechanism of three steps including both the conformation selection and induced fit was proposed for binding of Chz1 to H2A.Z-H2B.

## Materials and methods

### Equilibrium fluorescence measurements

Equilibrium fluorescence measurements were carried out on a cary eclipse fluorescence spectrophotometer (Agilent). Experiments were performed at T = 298 K in the same condition as kinetic experiments with the Trp139 excitation at 290 nm, and emission spectra were recorded from 300 to 450 nm. In the case of salt (urea) variation experiments, the mixing concentration of protein samples were held constant while additional salt (urea) concentration was gradually changed from 0.1 M to 0.7 M (1 M to 7 M). For the binding experiments between TChz and TBZ, TChz was titrated into 2 *μ*M TBZ by fixing salt/urea concentration at a particular point.

One significant factor, the inner filter effect (IFE) which was originated from the re-absorption of the emitted light in the excitation and emission regions can lead to underestimation of the fluorescence measurement signals. In solvent variation equilibrium fluorescence measurements, the protein concentrations were held constant and the re-absorption of salt and urea were negligible. While for the protein binding experiments, the IFE was prevented in each data points by subtracting baselines corresponding to different TChz concentrations.

### Circular dichroism spectroscopy

CD spectra were recorded using a J-820 (JASCO) spectropolarimeter at room temperature. A 3 mm path length cuvette was used, and far-UV spectra were recorded from 260 nm–200 nm with a scan speed of 50 nm/min. 5 *μ*M protein Samples were measured in 0.01 M PBS (pH 7.4) with blank subtracted. Each spectra was an accumulation of 3 repeated measurements.

### Isothermal titration calorimetry measurements

ITC was carried out with a NANO-ITC calorimeter (TA Instruments) at 298 K. Protein samples were extensively dialyzed and degassed before experiments. For each experiment, 200 *μ*M TBZ or TBZ_Y139W_ was drop wise injected into 20 *μ*M TChz. After subtracted baseline, the raw heat changes were obtained by integrating the areas under injection peak and were analyzed using the NANOanalyze software (TA Instruments) and replotted with Origin 8.5. The free energy change (Δ*G*), enthalpy change (Δ*H*) and entropy change (Δ*S*) were calculated according to the thermodynamic formulas of Δ*G* = −*RTlnK*_*A*_ = Δ*H*−*T*Δ*S*, Where *K*_*A*_ is the association constant at absolute temperature (T = 298 K) and R represents gas constant (8.3151 J mol^−1^ K^−1^).

### Stopped-flow fluorescence measurements

The kinetics of TChz and TBZ binding was measured using an upgraded J-820 Circular Dichroism Chiroptical Spectrometer (JASCO) with SFS-494 stopped-flow system as accessory instrument. Fluorescence signal was measured at T = 298 K, in 0.01 M PBS (pH 7.4). Measurements with high salt or urea concentrations were carried out also in 0.01 M PBS (pH 7.4) with different concentrations of additional NaCl and urea. Excitation was at 290 nm, and the fluorescence signal change was monitored by using a 320 nm long-pass cutoff filter (JASCO). Kinetic traces corresponding to different experiment conditions were recorded by varying the concentration of TChz while keeping the concentration of TBZ_Y139W_ constant at 1 *μ*M. Each obtained kinetic trace was an average of at least 5 individual injections. For analytical convenience, fluorescence signal was rearranged to set the starting fluorescence signal at 1 while keeping the amplitude unchanged.

### Free energy dependence calculation

The standard Gibbs free energy change in urea induced denaturation was shown to be linearly dependent on the denaturant concentration [41, 42]:

$$\begin{array}{c} \Delta G_{ND}=\Delta G_{ND}\left(H_{2}O\right)+m_{ND}^{urea}\left[urea\right] \end{array}$$ (1)

Where Δ*G*_*ND*_(*H*_2_*O*) represented the Gibbs free energy change in pure water, and $m_{ND}^{urea}$ was the slope of the free energy with respect to urea concentration. In the case of protein binding, a linear relationship was expected for the equilibrium association rate constant *K*_*A*_ and urea concentration. Since the equilibrium association constant *K*_*A*_ was related to standard Gibbs free energy change with Δ*G*_*binding*_ = *RTlnK*_*A*_, thus:

$$\begin{array}{c} lnK_{A}=\frac{\Delta G_{binding}\left(H_{2}O\right)}{RT}+\frac{m_{binding}^{urea}}{RT}\cdot \left[urea\right] \end{array}$$ (2)

At the midpoint of the conformation transition that half population of the native state was transformed to denatured state corresponding to an equilibrium constant *K*_*A*_ = 1, the standard Gibbs free energy change was equal to zero. Thus we have the following equation [43]:

$$\begin{array}{c} \Delta G_{ND}\left(H_{2}O\right)=-m_{ND}^{urea}{\left[urea\right]}_{1/2} \end{array}$$ (3)

On the other hand, the dependence of oppositely charged protein interaction Gibbs free energy on ionic strength could be described by electrolyte theory. It had been demonstrated that the association rate enhanced by electrostatic forces was approximated well by a transition state theory formula $k_{a}=k_{a}^{0}\left(-\Delta G_{el}^{‡}/k_{B}T\right)$ [44, 45]. $\Delta G_{el}^{‡}$ was the electrostatic interaction free energy in the reactive region, which was thought to form a virtual complex termed ‘transient complex’ [46]. Therefore if electrostatic potential contributed mostly in the rate-limiting step of association, the logarithm of association rate *lnk*_*a*_ was linearly related to logarithm of the activation free energy change of the association reaction due to electrostatic potentials. As a finite system, the assemble average over the transient complex configurations was dominated by the configuration with the lowest interaction energy. Such that, $\Delta G_{el}^{‡}=<U_{el}{>}^{‡}$. The average of the interaction energies was essentially the electrostatic potential change for bringing diffusive proteins together and was proportional to $RTln\gamma_{\pm }^{el}$ [47]. Where $\gamma_{\pm }^{el}$ was the electrostatic activity coefficient. Therefore, a formula could be obtained similar to urea denaturation reaction:

$$\begin{array}{c} \Delta G_{TD}^{‡}=\Delta G_{TD}^{‡}\left(H_{2}O\right)+m_{TD}^{salt}ln\gamma_{\pm }^{el} \end{array}$$ (4)

Where $\Delta G_{TD}^{‡}\left(H_{2}O\right)$ represented the standard Gibbs free energy change from denatured states to transient complexes in pure water, $m_{ND}^{salt}$ was the slope of free energy dependence. Given the mathematical complexity of accurate theory in high ion concentration, we had chosen a simplified approximation to quantify the ionic strength dependence of the rate constants by involving an extended Deby-Hückel formula [48].

$$\begin{array}{c} log\gamma_{\pm }^{el}=\frac{A|Z_{1}Z_{2}|\sqrt{I}}{1+Ba\sqrt{I}} \end{array}$$ (5)

Where $I=\frac{1}{2}\sum c_{i}Z_{i}^{2}$ was the ionic strength of the electrolyte and *Z*_1_ and *Z*_2_ were the charges of the electrolyte. for NaCl, *I* = [NaCl] and *Z*_1_ = *Z*_2_ = 1. The constants A and B were taken to be A = 0.510 mol^−1^ dm^3/2^ and B = 3.288 nm^−1^ mol^−1/2^ dm^3/2^ for water as solvent at 25°C [47, 49]. Parameter *a* = 4.62 Å was the distance of closest approach of the ions, a value corresponding to the sum of the radii of Na^+^ and Cl^−^ which includes the first hydration layer that were immobilized by electrostatic interactions [50]. The relative small amount of phosphate ions present in the buffer was neglected in the calculation. A similar linear relationship for electrolyte can be obtained on the same basis of urea case:

$$\begin{array}{c} lnK_{A}=\frac{\Delta G_{TD}^{‡}\left(H_{2}O\right)}{RT}+\frac{m_{TD}^{salt}}{RT}\cdot ln\gamma_{\pm }^{el} \end{array}$$ (6)

And for salt at the midpoint of state transition for initial diffusion states to transient complex states:

$$\begin{array}{c} \Delta G_{TD}^{‡}\left(H_{2}O\right)=-m_{TD}^{salt}ln\gamma_{\pm ,1/2}^{el} \end{array}$$ (7)

Eqs (1)–(7) were used for the equilibrium and kinetic analysis of free energy dependence of urea and NaCl, respectively, by assuming the linear relationship hold for specific region of ion strength.

### Numerical fitting and parameter space analysis

Since two phases were experimentally observed, the minimum model that we could employ in this study corresponding to the binding of TChz with TBZ_Y139W_ was a two-step wise reversible reaction.

Generally, three types of two-step wise models corresponding to three distinct binding-folding pathways were used to evaluate the microscopic reaction rates:

Conformation-selection:

case (I):

$$\begin{array}{c} \left[\text{TChz}\right]\overset{{\text{k}}_{1+}}{\underset{{\text{k}}_{1-}}{⇌}}{\left[\text{TChz}\right]}^{*}+\left[\text{TBZ}\right]\overset{{\text{k}}_{2+}}{\underset{{\text{k}}_{2-}}{⇌}}\left[\text{TChz}-\text{TBZ}\right] \end{array}$$ (8)

case (II):

$$\begin{array}{c} \left[\text{TBZ}\right]\overset{{\text{k}}_{1+}}{\underset{{\text{k}}_{1-}}{⇌}}{\left[\text{TBZ}\right]}^{*}+\left[\text{TChz}\right]\overset{{\text{k}}_{2+}}{\underset{{\text{k}}_{2-}}{⇌}}\left[\text{TChz}-\text{TBZ}\right] \end{array}$$ (9)

Induced-fit:

$$\begin{array}{c} \left[\text{TChz}\right]+\left[\text{TBZ}\right]\overset{{\text{k}}_{1+}}{\underset{{\text{k}}_{1-}}{⇌}}{\left[\text{TChz}-\text{TBZ}\right]}^{*}\overset{{\text{k}}_{2+}}{\underset{{\text{k}}_{2-}}{⇌}}\left[\text{TChz}-\text{TBZ}\right] \end{array}$$ (10)

Nevertheless, a mixed model that had three reaction steps was also proposed for the interpretation of stopped-flow kinetic data.

Mixed model:

$$\begin{array}{c} \left[\text{TChz}\right]\overset{{\text{k}}_{1+}}{\underset{{\text{k}}_{1-}}{⇌}}{\left[\text{TChz}\right]}^{*}+\left[\text{TBZ}\right]\overset{{\text{k}}_{2+}}{\underset{{\text{k}}_{2-}}{⇌}}{\left[\text{TChz}-\text{TBZ}\right]}^{*}\overset{{\text{k}}_{3+}}{\underset{{\text{k}}_{3-}}{⇌}}\left[\text{TChz}-\text{TBZ}\right] \end{array}$$ (11)

This mixed model was composed with the first step being second order reaction while the later step being conformation rearrangement of TChz was not possible since the fluorescence intensity would change once TChz had contacted with TBZ_Y139W_.

As discussed in the result section, our data were analyzed with Eq (10). The ordinary differential equations corresponding this mechanism were used to do the nonlinear least-square fit of the stopped-flow fluorescence signal by assuming that the microscopic rates (*k*_1+_, *k*_1−_, *k*_2+_ and *k*_2−_) were constants independent of reactant concentration and temperature. To simplify data analysis, the fluorescence signal was normalized by setting the initial data point equals to 1. In other words, the fluorescence intensity of unbound TBZ_Y139W_ was normalized to unit. With this experimental setup, the total fluorescence intensity was described by the following equation:

$$\begin{array}{ccc} F & = & 1-F_{I}\times {\left[\mathrm{TChz}-TBZ_{Y139W}\right]}^{*}\\ & & +F_{B}\times \left[\mathrm{TChz}-TBZ_{Y139W}\right] \end{array}$$ (12)

with the amplitude of fluorescence signal of intermediate *F*_*I*_, the bound state *F*_*B*_, and the microscopic rate constants as the fitting parameters.

The parameter space analysis was based on two-step induced-fit model as demonstrated by Eq (10). The kinetic data obtained from stopped-flow fluorescence signal of various protein concentration ratios were fitted with one set of constant kinetic parameters. The kinetic rate constant pair being estimated were held still to the grid point of the parameter space while allowing the other parameters for nonlinear least-square fitting to the kinetic data. The error squares were summed to give a ratio of *SSE*_*min*_/*SSE*_*xy*_ which was plotted with respect to the kinetic rate constant pair.

Fitting errors of each kinetic rate constant are obtained by setting the threshold to be *SSE*_*min*_/*SSE*_*xy*_ = 0.8 [51], and the percentage ranges are computed as (*k*_*upper*_ − *k*_*lower*_)/2*k*_*min*_.

## Results and discussion

### Validation of tryptophan variant of H2A.Z-H2B

**Fig 1:**
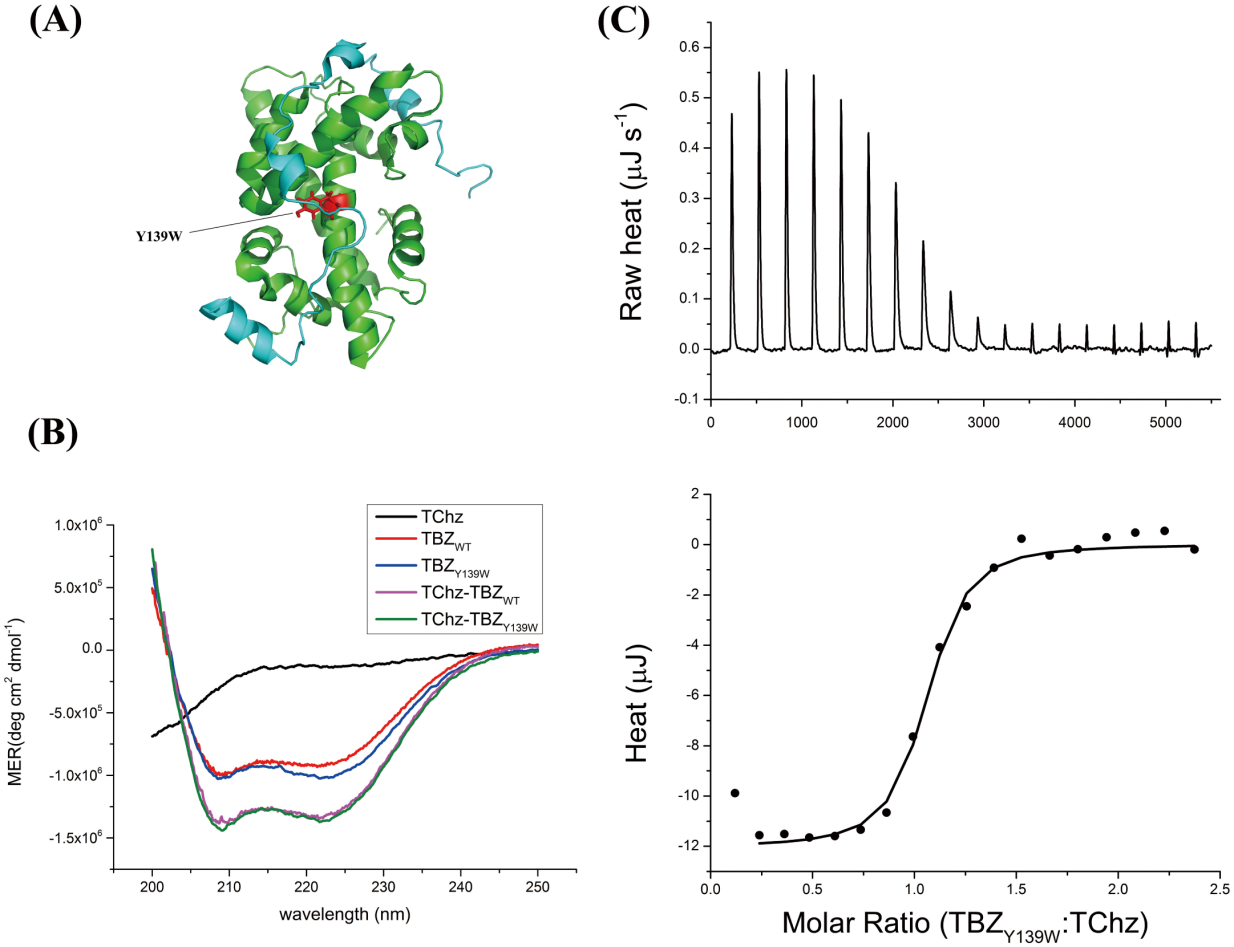
Pseudo-WT mutant of histone heterodimer TBZ. (A) Cartoon structure of the TChz-TBZ complex (Protein Data Bank (PDB) code 2JSS). TChz was shown in cyan and TBZ in dark green with the mutation site shown in red sticks. Paragraph was generated with Pymol. (B) Far-UV CD spectra of TChz_WT_ (black), TBZ_WT_ (red), TBZ_Y139W_ (blue), TChz-TBZ_WT_ (magenta) and TChz-TBZ_Y139W_ (olive) with protein concentration ratio of 1:1.05. (C) Binding thermodynamic measurement of TChz interacting with TBZ_Y139W_ measured by ITC. The integrated heat was fitted to a 1:1 isothermal binding model, shown in lower picture with solid line. Experiments were performed at 298 K as described in Materials and Methods.

Protein intrinsic fluorescence greatly facilitates the usage of spectroscopy methods to study the binding kinetics of protein interaction with high sensitivity. However, the truncated protein recognition fragments of TChz and TBZ contain only Tyr residues that will require high concentration (> 10 *μ*M) protein samples to obtain reliable fluorescence signal change with stopped-flow techniques. Given the stereochemical and physical similarity of Tyr and Trp, the Tyr139 residue (Fig 1A) was chosen as the label site to be substituted to Trp. The validation of the Trp variant was then evaluated based on structural and energetic similarity using CD spectroscopy (Fig 1B) and ITC (Fig 1C). The far-UV CD spectra of TBZ_Y139W_ was very similar to the WT protein, suggesting the secondary structure was not greatly altered due to this mutation. Furthermore, the binding of TChz to TBZ_Y139W_ increasesed the CD signal, meaning that the binding forced TChz to change from complete disordered state to a more structured state, just as what the WT TBZ did. Moreover, the enthalpy (Δ*H*) and entropy (Δ*S*) changes calculated by fitting a 1:1 isothermal model to ITC titration date was -34.06 ± 0.4 kJ mol^−1^ and 14.16 J mol^−1^ K^−1^, respectively, with the stoichiometric number determined as *n* = 0.97. Thus the dissociation rate constant was derived to be 0.33 ± 0.02 *μ*M at 310 K, a value very similar to previous published results for the wild type, 0.10 ± 0.01 *μ*M [33]. Therefore, the mutant TBZ_Y139W_ was set to be the pseudo-WT protein of TBZ.

The free energy change, which can be obtained from Δ*G* = Δ*H* − *T*Δ*S*, was calculated to be -38.28 KJ mol^−1^. On the other hand, the simulation results of binding between Chz1 and H2A.Z-H2B by structure-based coarse-grained model which implemented Debye-Hückel model obtained a typical 3-state binding transition with the first free energy barrier of 4.4 KT (K as the Boltzman cosntant and T as the absolute temperature) [34]. The simulation was performed near the transition temperature where dissociation and association states had almost the same possibility in order to sample more configurations around the transition state. Therefore, the thermodynamic simulations were performed at a higher temperature and protein concentration as compared with experimental dilute solution conditions. However, we can still estimate the theoretical calculated free energy change from the height of transition state barrier. In macroscopic scale systems, we should replace KT with RT (R as the gas constant) and led to 10.91 KJ mol^−1^ which was on the same order of experimentally determined free energy change. Besides, from the characteristic signs of the enthalpy and entropy changes, we can obtain insights of the driven forces of protein association. The binding of Chz1 to H2A.Z-H2B was driven by both enthalpy and entropy changes given that Δ*H* and Δ*S* both favored association. Since the sign of enthalpy change was negative and the sign of entropy change was positive, we can conclude electrostatic forces contributed mostly for Chz1 and H2A.Z-H2B association [52]. Furthermore, as the stoichiometric number determined by ITC was very close to 1 and the 1:1 isothermal model fitted well to the ITC titration raw heat data, the binding of Chz1 to H2A.Z-H2B is not a cooperative process. Therefore, we could safely exclude more complicated mechanism that involved with cooperative interactions.

### Equilibrium dissociation of Chz1 from H2A.Z-H2B is a two-state process

Histone chaperone Chz1 was an intrinsically disordered protein which has preference for histone variant H2A.Z. The interaction of Chz1 with H2A.Z-H2B formed a heterotrimer complex which further stabilized the association of the histone dimer. In the nucleosome, the H2A.Z-H2B dimers have extensive contacts with DNA. As the result, H2A.Z-H2B and Chz1 were highly charged. The NMR study revealed the Chz1-H2A.Z-H2B heterotrimer was mainly stabilized by the electrostatic rather than hydrophobic interactions [40]. Relaxation dispersion NMR spectroscopy also exhibited that the presence of electrostatic forces led to a higher association rate beyond the diffusion limit [33]. Therefore, in purpose of revealing the roles electrostatic and hydrophobic forces played in the complex formation of Chz1-H2A.Z-H2B heterotrimer, equilibrium salt/urea induced denaturation experiments were performed using both fluorescence and CD spectroscopy methods. As the Trp mutation site was located in the middle valley of the interaction complex, a rather hydrophobic micro-environment, both fluorescence intensity and maximum wavelength change could be used to give information about the complex structure change upon the increasing of salt/urea concentration.

As shown in S1 and S2 Figs, both the fluorescence intensity and maximum emission peak wavelength (*λ*_*max*_) shift of Chz1 dissociation from TBZ_Y139W_ along with increasing NaCl or urea could be fitted to a two-state model. The fluorescence changes in different solvent conditions of TBZ_Y139W_ could be attributed to the energy transfer of Trp139 to its nearest neighborhood amino acids as explained in S1 File. The binding of TChz increased the fluorescence intensity of TBZ_Y139W_ by modulating the structure of TBZ_Y139W_ and decreased the energy transfer of Trp139 to its neighborhood amino acid acceptors.

NaCl and urea had different impacts on the dissociation process. Increasing concentration of NaCl led to more compact structure of TBZ_Y139W_, while urea induced conformation change tended to be looser. The maximum emission peak wavelength (*λ*_*max*_) shift of NaCl induced dissociation from TBZ_Y139W_ was quite different from the urea induced emission peak shift. Since the emission peak position of tryptophan was largely determined by the surrounding micro-environment, the reciprocal behavior here indicated the dissociation reaction was not a simply one-step process. As explained in the S1 File, this observed phenomenon could be attributed to the minor motions of the middle motif of TChz with additional NaCl presented in the solution. The thermodynamic parameters derived from the experiments were shown in Table 1 based on an equilibrium two-state model as described in the S1 File. Noted that the results from fitting to CD spectra were inaccurate because the CD signal change were very small compared to protein native state CD signals. Besides, the obtained free energy change from fitting CZB fluorescent intensity and *λ*_*max*_ shift data showed relatively large diverge from the ITC results. This inconsistence was an indicator of the failure of the two-state equilibrium association model caused by the interplay of beforehand dissociation of TChz middle motif and the specific location of the labeled Trp139 fluorophore. However, the dissociation of TChz from TBZ_Y139W_ at equilibrium condition was rationally fitted by a two-state model as shown in urea-induced fluorescence/CD and NaCl-induced CD signal changes while the free energy changes were all comparable to ITC results -38.28 kJ mol^−1^ at 298 K. Taken together, these results indicated the dissociation of TChz from TBZ at equilibrium condition can be well described as a two-state process.

**Table 1:** Comparison of thermodynamic parameters for NaCl- and urea-induced conformation change of TChz-TBZ_Y139W_ (CZB) in 0.01M PBS (pH 7.4), at 298K^a^.

NaCl	Observed Property	Δ*G*_*ND*_(*H*_2_*O*) (KJ mol^−1^)	$m_{ND}^{salt}$ (KJ mol^−1^)	$ln\gamma_{\pm ,1/2}^{el}$
*CZB*^b^	CD (222 nm)	-27.00 ± 0.59	69.78 ± 1.44	0.39
	Fluorescence Intensity	-57.76 ± 14.05	147.51 ± 36.05	0.39
	*λ*_*max*_ shift	-49.83 ± 7.40	128.47 ± 19.00	0.39
TBZ		nd^c^	nd	nd
Urea	Observed Property	Δ*G*_*ND*_(*H*_2_*O*) (KJ mol^−1^)	$m_{ND}^{urea}$ (KJ mol^−1^ M^−1^)	[*Urea*]_1/2_ (M)
*CZB*^b^	CD (222 nm)	-35.55 ± 3.83	9.23 ± 0.99	3.85
	Fluorescence Intensity	-30.48 ± 4.01	8.60 ± 1.14	3.54
	*λ*_*max*_ shift	-22.90 ± 1.84	5.75 ± 0.46	3.98
TBZ^b^	CD (222 nm)	-18.33 ± 0.53	5.54 ± 0.16	3.31
	Fluorescence Intensity	-32.97 ± 5.58	9.84 ± 1.64	3.35
	*λ*_*max*_ shift	-32.96 ± 3.50	9.79 ± 1.03	3.37

^*a*^ Evaluation was based on equilibrium two-state model.

^*b*^ Excitation wavelength 290 nm and emission wavelength 340 nm.

^*c*^ nd represents not determined data.

### Equilibrium free energy dependence

**Fig 2:**
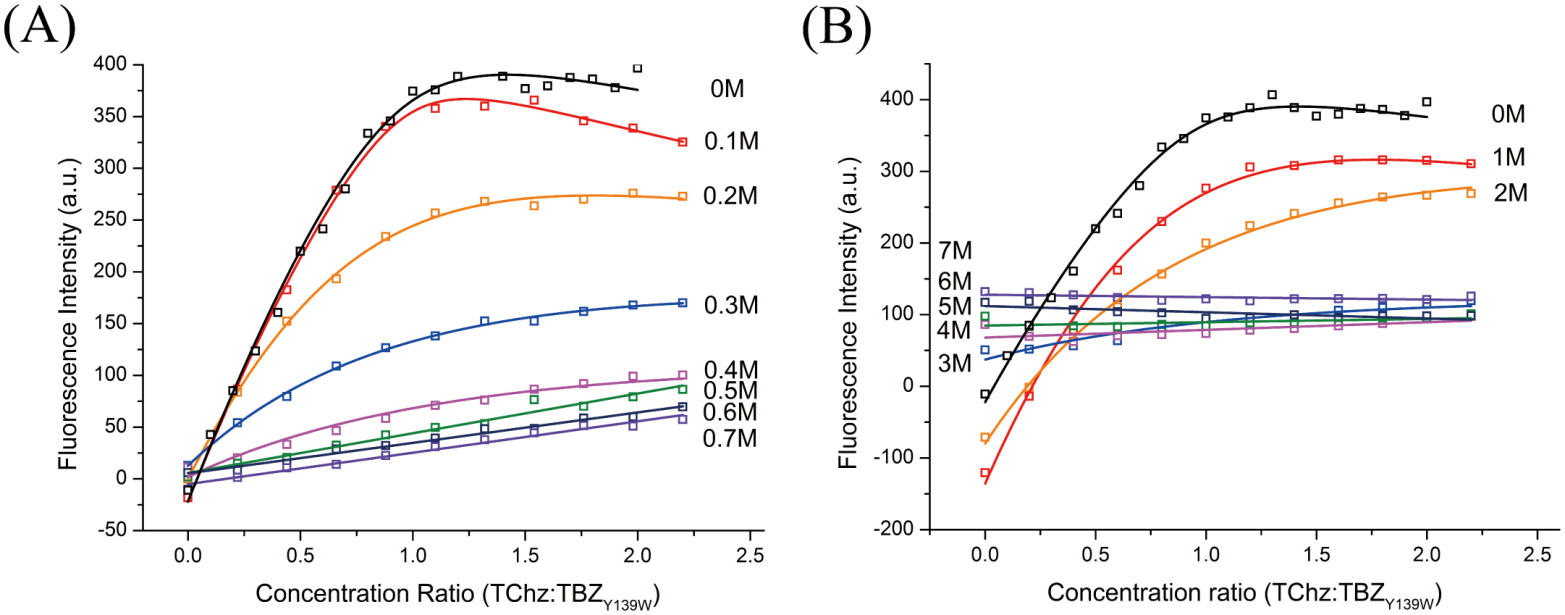
Equilibrium fluorescence titration of TChz binding to TBZ_Y139W_. Experiments were performed by titrate 20 *μ*L 30 *μ*M TChz into 800 *μ*L 5 *μ*M TBZ_Y139W_ in 0.01 M PBS (pH 7.4), at 298 K. The artificial fluorescence changed caused by decrease of TBZ_Y139W_ concentration was corrected by subtracting baseline of blank titration control experiment. Data of titration of TChz into TBZ_Y139W_ in varies NaCl concentration (A) and titration of TChz into TBZ_Y139W_ in varies urea concentration (B) are plotted with the best fitting to an equilibrium two-state model as described in the supporting information.

**Fig 3:**
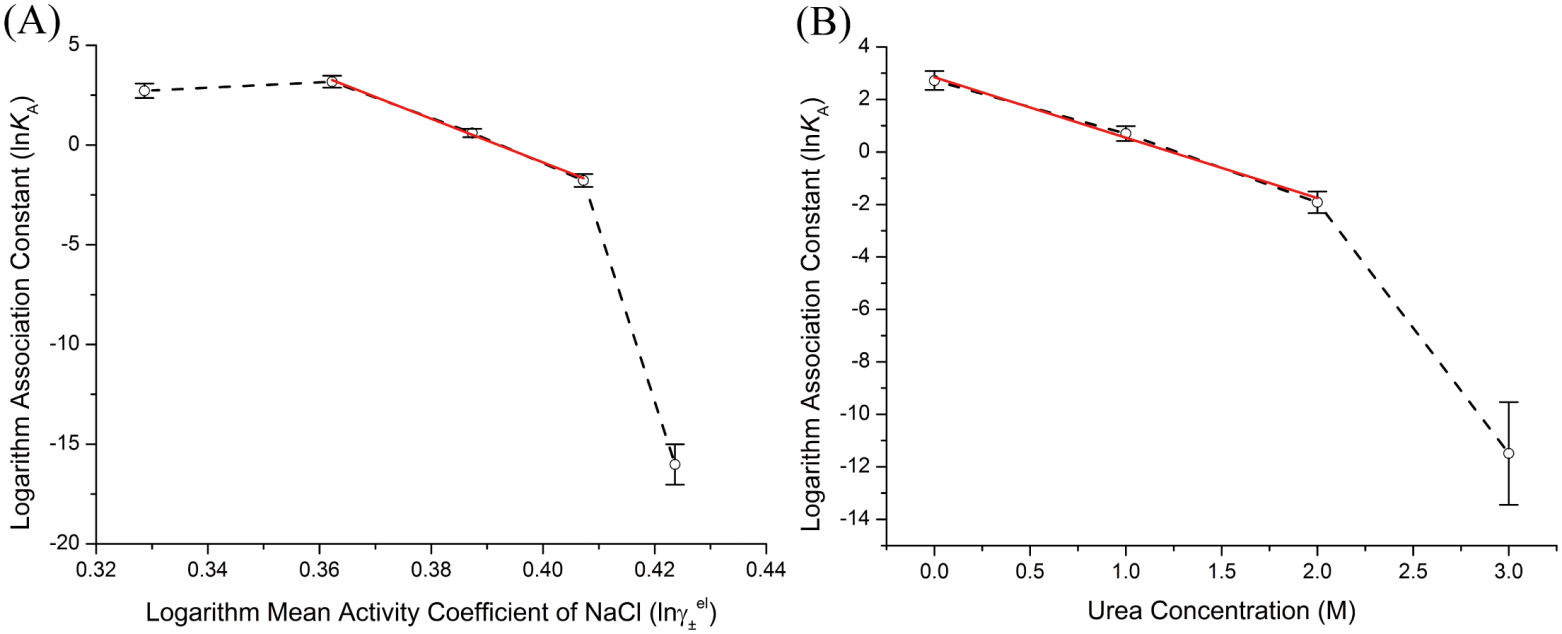
Equilibrium association constants dependence analysis. Dependence of equilibrium association constants on (A) NaCl mean activity coefficient and (B) urea concentration. The equilibrium association constants are referred to open symbols and represented with their logarithm values. Solid wine lines are the linear best fits according to Eqs (2) and (6).

For further characterization of the free energy change along NaCl/urea concentration, the equilibrium dissociation rate constants with varied concentrations of NaCl/urea were determined by fluorescence titration experiments. As seen in Fig 2, the fluorescence signal at 335 nm exhibited a two-state behavior and was well fitted to the two-state transition model as described in S1 File and the fitting results were represented in Fig 3. However, the equilibrium two-state model failed when NaCl concentration > 0.4 M or urea concentration > 2 M, suggesting specific interactions were disrupted by NaCl and urea beyond these denaturant concentration.

The linear fitting results were shown in Fig 3, the calculated slopes which corresponding to the free energy dependence were listed in Table 2. Note that the initial point of Fig 3(A) did not obey the linear relationship of Eq (6) which showed a discontinuous transition of the association constant dependence. The main effect of NaCl presence in solution was to screen the long range forces of electrostatic interactions, such as H-bond, salt bridge, etc. For an electrostatic dominant system where electrostatic interactions contributed the most to protein association stability, one would expect linear free energy dependence with mean activity coefficient. Therefore the nonlinear to linear transformation of free energy dependence shown in Fig 3(A) could be rationalized by the participating of short range hydrophobic forces, i. e., the dissociation of TChz from TBZ_Y139W_ was a two-step process. The free energy dependence on urea was essentially linear for urea concentration less than 2 M. The presentation of urea in solvent has effects on proteins through either direct way (denaturant binding model) or indirect way (solvent exchange model) [53]. Urea can bind to protein directly by forming H-bonds to exposed amide groups on the protein backbone that were stronger than the amide-amide H-bonds of the associated protein heterodimer. Alternatively, urea may also act indirectly by changing the water structures and dynamics, reducing the solution hydrophobic effect and promoting the exposure of the hydrophobic residues to solvents. From either point of view, the linear dependence observed in Fig 3(B) indicated the conformation change of the protein heterodimer caused by urea denaturation was a two-state process. The free energy dependence obtained by urea induced fluorescence change was -5.75 ± 0.42 kJ mol^−1^ M^−1^, which was significantly less than the free energy dependence of NaCl induced fluorescence change, -272.10 ± 11.97 kJ mol^−1^. These results were consistent with the NaCl/urea concentration variation experiments. Therefore, from Fig 3(A) and 3(B) we demonstrated that hydrophobic forces had minor influence of the protein free energy change, while electrostatic forces persisted until large conformation happened and was the dominant interactions. Noted that electrostatic forces were also the main contribution factor for binding stability [40].

**Table 2:** Equilibrium and kinetic rate constants dependence on NaCl and urea concentration^a^.

	$m_{ND}^{salt}$ (KJ mol^−1^)	$m_{ND}^{urea}$ (KJ mol^−1^ M^−1^)
*K*_*A*_^b^	-272.10±11.97	-5.75±0.43
*k*_1+_	-86.24±7.61	-0.22±0.08
*k*_1−_	42.39±7.56	0.25±0.17
*k*_2+_^c^	-2.78±0.09	21.17±11.98
*k*_2−_	2.65±3.64	0.02±0.01

^*a*^ Kinetic rate constants extracted from fitting the stopped-flow kinetic data to a three-state induced-fit model.

^*b*^ Equilibrium association constant dependence was obtained by fitting to Fig 3 with Eqs (2) and (6).

^*c*^ Fit to an exponential decay equation, the decay constants were listed.

### Kinetic characterization of TChz binding to TBZ

Protein association often exhibits a multi-step mechanism along the topography of underlying complex energy landscape. As protein binding reactions are effectively reversible, the stepwise reaction rate constants are coupled together to determine the time evolution of the final product concentration which is often labeled as the reported state. With this complication, the effort to obtain an analytical result through rate equations is not practical except for one step reactions. The arguably most accurate approach used extensively for estimating multi-step kinetic rate constants is the pseudo-first-order approximation. As a linearization first order approximation, the pseudo-first-order condition is created by providing an excess (above 10 folds) of one to other of the reaction components and setting the excess species concentration as constant throughout the kinetic analysis. The kinetic data is fitted to a sum of decoupled exponential terms for each step of the reaction. In this way, the rate constants are extracted for a number of protein ratios for reducing errors. However, a theoretical pseudo-first-order condition is often hard to be achieved in experiments. Therefore, in this work we chose a straightforward numerical integration method to extract reaction rate constants of TChz binding to TBZ from experiments.

**Fig 4:**
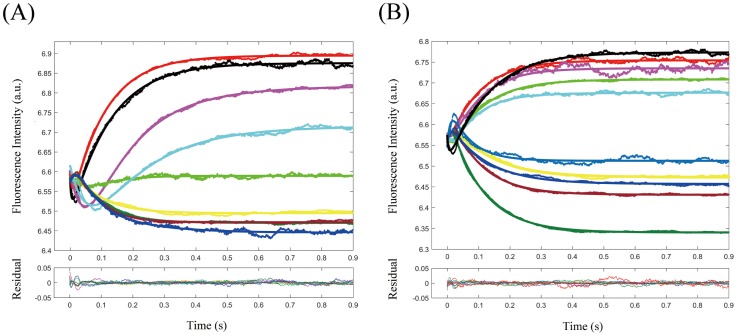
Stopped-flow fluorescence signal of TChz binding to TBZ_Y139W_ in various NaCl (A) and urea (B) concentration. The binding experiment conditions are referred to colored lines in (A): *black*, PBS; *red*, 0.5 M NaCl; *magenta*, 0.1 M NaCl; *green*, 0.15 M NaCl; *cyan*, 0.2 M NaCl; *yellow*, 0.3 M NaCl; *oliver*, 0.4 M NaCl; *wine*, 0.5 M NaCl; *blue*, 0.6 M NaCl. In (B): *black*, PBS; *red*, 0.5 M urea; *magenta*, 1 M urea; *green*, 1.5 M urea; *cyan*, 2 M urea; *yellow*, 3 M urea; *oliver*, 4 M urea; *wine*, 5 M urea; *blue*, 6 M urea; *navy*, 7 M urea. The fitting residuals corresponding to each kinetic traces are collectively plotted in the below pictures. Experiments were performed by mixing 10 *μ*M equimolar TChz and TBZ at 0.01 M PBS at 25°C.

The stopped-flow fluorescence signal were plotted with variant NaCl and urea concentrations in Fig 4. The kinetic traces exhibited an obvious two phase behavior with the first phase in the time regime of 0–10 ms and the second phase spanning from 10 ms to equilibrium (physiological condition). With NaCl concentration < 0.3 M, the first phase of fluorescence signal was decreased with the time course of binding reaction, indicating that with the accumulation of the kinetic intermediate fluorescence intensity was decreased. Since the fluorescence intensity change of TBZ_Y139W_ was mainly due to the energy transfer of Trp139 to its neighborhood amino acids, and the formation of this kinetic intermediate was not influenced by high ionic strength or urea concentration, this formation of binding intermediate was very likely due to the nonspecific electrostatic attraction of TChz with TBZ_Y139W_. By comparing the equilibrium and kinetic fluorescence signal under the same solvent conditions, we had reasons to believe, at NaCl concentration < 0.3 M, the emergent intermediate had similar conformation ensemble with the equilibrium states perturbed by NaCl. As a matter of fact, the fluorescence change of Trp139 only reflects its very neighborhood micro-environment alter since energy transfer efficiency was inversely proportional to *r*^6^. Thus the decrease of fluorescence intensity by a different conformation ensemble while TBZ_Y139W_ having little structure change was not likely to occur. Also, it was interesting to see that the first phase fluorescence signal was increased in high NaCl/urea concentration. At high NaCl/urea concentration, as the fluorescence and CD spectra shown, TBZ_Y139W_ had transformed to molten globule state. The nonspecific electrostatic interactions presence in the intermediate state might induce the conformation to less compact states, pulled the energy transfer acceptor away from Trp139, thus led to a small fluorescence intensity increase in the intermediate state.

The fluorescence signal obtained by stopped-flow experiments could be fitted to a two-step association model, such as Eqs (8)–(10) indicated. However, the pure conformation selection scheme was ruled out due to the following reasons: (*i*) The fluorephore was located in the valley of TBZ_Y139W_, and the conformation change of TChz could not result in a fluorescence decreased intermediate, thus Eq (8) was not suitable for modeling the binding of TChz to TBZ_Y139W_; (*ii*) The fluorescence signal change might be due to the accumulation of TBZ_Y139W_ in a less fluorescent state, hence be selected by solvent presence TChz. However, the concentration dependent experiment showed an accelerate behavior of the fraction maximum location of intermediate species along time course of the binding-folding reaction, which was not expected if Eq (9) was applied. In the case of Eq (9), with increasing of TChz concentration, the second step of binding-folding reaction was accelerated with the excess of TChz in solvent; (*iii*) The intermediate revealed by spectra and free energy dependence analysis showed that the first step of association was governed by electrostatic potentials, but in the case of Eq (9) electrostatic interactions will not create a conformation switch without the involvement of TChz; (*iv*) As seen from Fig 4, the intermediate emerged even in high NaCl/urea concentration condition, but a conformation ensemble with increased fluorescence intensity was not likely to appear in the molten globule state of TBZ_Y139W_. Nevertheless, the analysis here could not exclude a mixing model with initial conformation selection of TChz as Eq (11) demonstrated.

The fitting results of TChz binding to TBZ_Y139W_ to a two-step induced-fit model were collectively shown in Fig 4 with colored solid lines representing the best fit. Fitting residuals were collectively plotted in the pictures below. It should be noted that a significant part of the first phase was shaded by the stopped-flow dead time, therefore the fitting to first phase was not quite accurate with relatively large residuals.

The association rate constant *k*_1+_ had the order of 10^7^ M^−1^ s^−1^, which was an order magnitude less than the NMR relaxation dispersion results [33]. Nevertheless, the rate-limiting step was the second step by having the rate constant on the order of 10 s^−1^. While in the NMR relaxation dispersion experiments, no kinetic intermediate was observed. The possible reason to this diversity may be due to that structure of the intermediate was similar to the bound state of TChz-TBZ, making the second conformation rearrangement step be attributed to the random fluctuations of the middle long irregular structure for TChz in the NMR relaxation dispersion experiment. The reason that this conformation rearrangement could be detectable in our study was because the position of reporter fluorephore was located in the valley of middle motif of TBZ_Y139W_, which was thought to be the last step of the conformation rearrangement processes.

**Fig 5:**
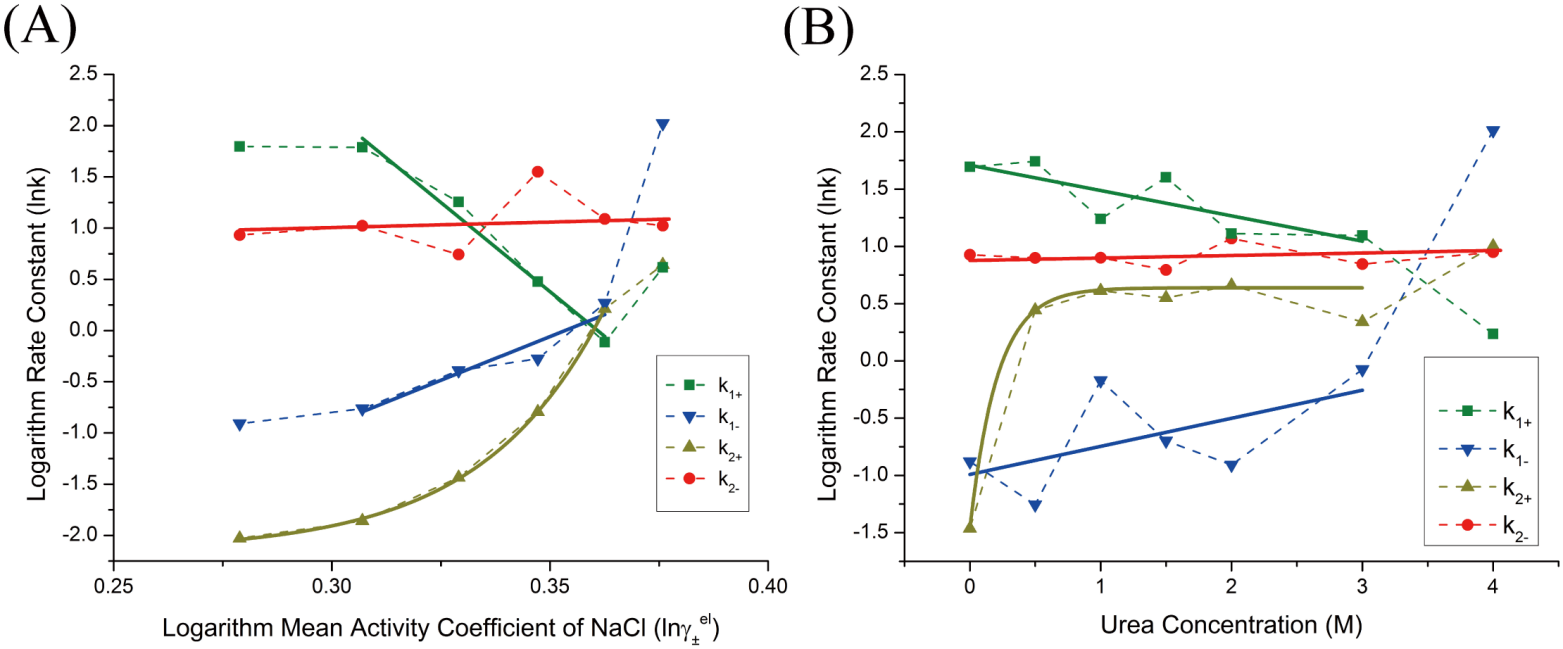
Kinetic parameters obtained from nonlinear least-square fitting of fluorescence intensity change of TChz binding to TBZ_Y139W_ with a two-step induced-fit model. (A) Rate constants dependence on NaCl mean activity coefficient. (B) Rate constants dependence on urea concentration. Kinetic rate constants were plotted in their logarithm form with colored points: *green square*, *k*_1+_ (s^−1^ *μ*M^−1^); blue inverted triangle, *k*_1−_ (s^−1^); *dark yellow triangle*, *k*_2+_ (s^−1^); *red circle*, *k*_2−_ (s^−1^). The black solid line were best linear fittings of the rate constants in correspond to the linear region that were characterized by equilibrium experiments.

The kinetic rate parameter dependence on NaCl mean activity coefficient and urea concentration were analyzed and fitted to linear or exponential equations (Fig 5). The fitting results were listed in Table 2. The microscopic kinetic rate constants were determined by the molecular interactions of the associated proteins under the modulation of solvent molecules and were reflected through the molecular population changes to give continuous fluorescence signals during association. Therefore, the different microscopic kinetic rate constants indicated the differences in time evolution functions of the molecular populations and were closely related to the protein recognition mechanisms. In fact, the molecular populations can be viewed as the states possibility that a protein stayed during the association reaction. Thus the mechanism discussed in our bulk experiments also indicated the molecular association mechanism. The binding rate constant *k*_1+_ and *k*_1−_ exhibited a linearly dependence on mean activity coefficient of NaCl, which was consistent with equilibrium free energy dependence analysis. As seen in Fig 5, the initial point of TChz binding to TBZ_Y139W_ violated the linear relationship. The basis of free energy linear dependence rule relied on the assumption that protein diffusion from diffusive states to transient complex states was the rate-limiting step which was dominated by electrostatic forces. Therefore the invalidation of linear relationship indicated that an extra step of reaction other than diffusion was exist or the binding pathway was shifted due to increasing ion strength. As shown by Eq (11), if an initial conformation selection step was involved as suggested, the rate-limiting step might be changed from electrostatic aid association step to the TChz conformation selection step. On the other hand, if the initial conformation selection step was not present, the protein binding-folding reaction might have been shifted from a hydrophobic determined pathway to a more electrostatic dominant one with increase of ionic strengths. Indeed, both these two situations were proposed in the simulation study on Chz.core to H2A.Z-H2B [34]. However, since the structure of H2A.Z-H2B linked complex was not much changed with the addition of NaCl, an entire pathway shift would not be possible for this system. Therefore, we believed the binding of TChz to TBZ was a three-step reaction that involve both the conformation selection and induced-fit mechanism.

**Fig 6:**
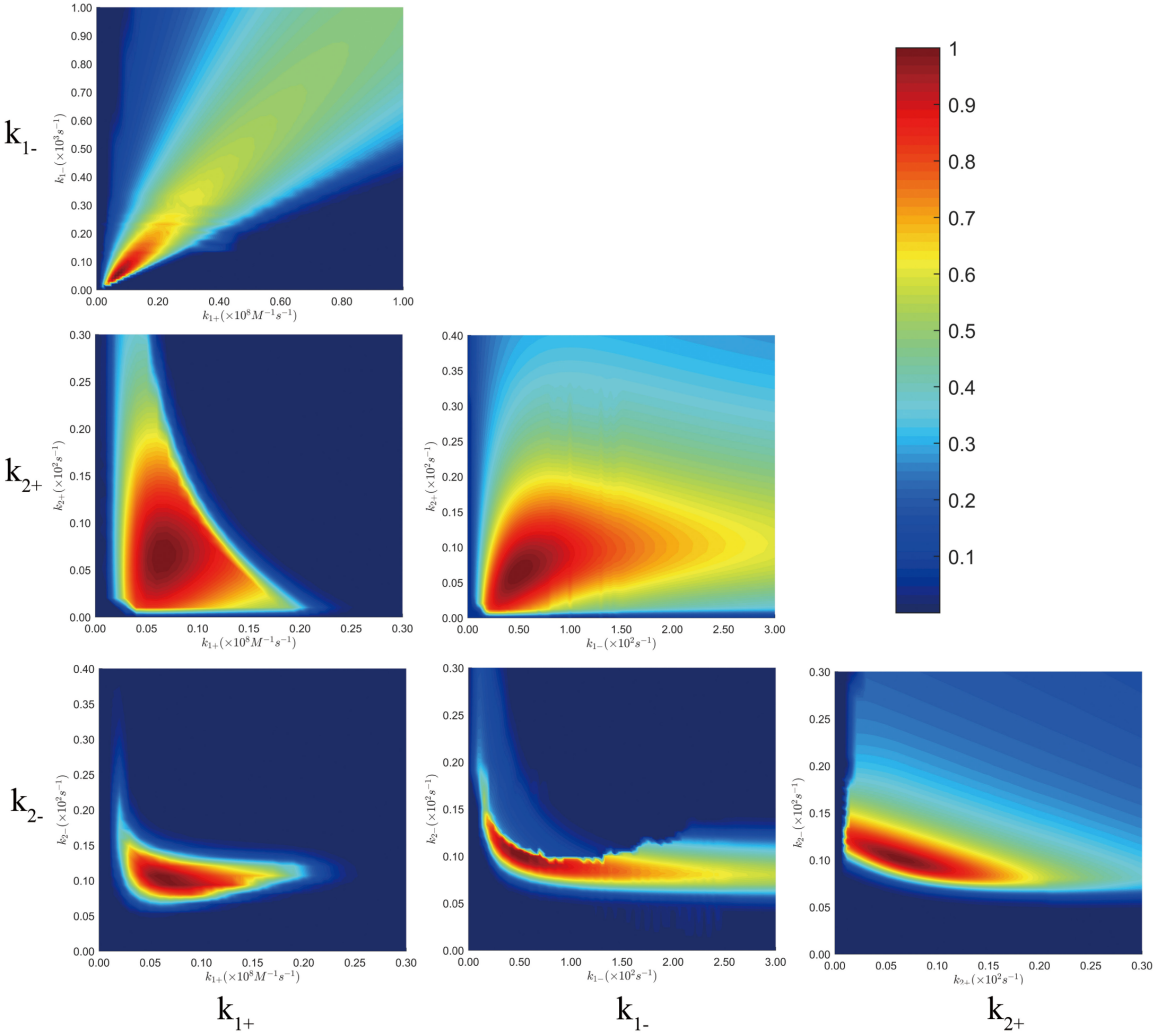
2-D parameter space plotted corresponding to pair-wise kinetic rate constants. The colors shown in color bar indicated the degree of each kinetic rate constants pair approaching the minimum SSE which is normalized to unit. Analyzing was based on the two-state induced-fit model. Experiments were performed at 0.01M PBS at 25°C, and the mixing concentrations for TChz were set to be 4, 6, 8, 12, 20*μ*M, and TBZ_Y139W_ concentration is set constant to be 2*μ*M.

The second step rate constant *k*_2+_ in NaCl and urea induced kinetic experiments were exponentially dependent on mean activity coefficient and urea concentration with the dissociation rate constant *k*_2−_ almost a constant value throughout the experiments. Currently we did not find a theoretical model to describe this kind of kinetic rate constant dependence thus the rate constant dependence was fitted with an exponential decay equation and the decay constants were listed in Table 2. The kinetic traces of TChz to TBZ_Y139W_ were discontinuously changed to a different pattern with high NaCl/urea concentration (Fig 5) and the rate constants remained unchanged with increasing ionic strengths and urea concentration within experimental error (Fig 6). This indicates the interaction of TChz with TBZ_Y139W_ in high NaCl/urea concentration was nonspecific which was consistent with the equilibrium spectra analysis. Note that the equilibrium association rate constant dependence was significantly larger than the kinetic rate constants dependence, indicating that the presence of excess ionic strength or denaturant influence the overall protein association/dissociation reaction and was more sensitive to the solvent condition change than individual microscopic rate.

### Parameter space analysis of kinetic data global fitting

Extracting kinetic microscopic rate constants by fitting the kinetic data directly to a model based on numerical integration is hopefully the best method in kinetic data analysis. Compared to analytical methods that are based on the simplified assumptions, numerical methods are more straightforward and have several advantages as compared with the traditional kinetic analyzing methods based on simplified assumptions such as pseudo-first order approximation. For example, the reaction rate constants can always be obtained if a model is proposed, while for pseudo-first-order approximation, the equation for extracting parameters from exponential fitting is often very complex for a reversible multistep reaction. Besides, fitting experimental data to multiple exponential functions fails to estimate the signal amplitudes of each reaction species. Moreover, even the excess species concentration is 10 times over other components, there is still about 10% uncertainty by setting one reactant concentration as constant. Despite the advantages, numerical methods have its shortcomings on revealing the relationship of multiple parameters with the complex relationships kinetic data. As a matter of fact, for a direct integration based on the differential equations, generally a larger derived set of fitting parameters has to take part in the numerical integration which leads to the question whether or not these fitting parameters are constrained by the measured kinetic data. This is also the reason of the failure of conventional error analysis. For these reasons, it is often too easy to assume an overly complex analytical model. Here we analysis the fitting parameter space [51] by constructing a 3-D confidence contours to estimate the fitting errors and demonstrate our model was essentially constrained by the experimental data.

The calculated 3-D confidence contour corresponding to the multidimensional parameter space was plotted with colors that represented the degree of each parameter pair approaching the minimum SSE (sum of squared errors of prediction) in Fig 6. And the percentage range for each kinetic data constants were listed in Table 3 with the best fitting parameters for various protein ratio experiments. As seen from the 3-D confidence contour, the fitting was converged to certain point and errors were within 10% for all the kinetic data point. It had to be mentioned that kinetic rate constants *k*_1+_ and *k*_1−_ were less constrained by the kinetic data, the reason was due to the shading of first phase fluorescence signal by stopped-flow dead time in the physiological condition.

**Table 3:** Best fit parameters and percentage range for parameter space analysis.

	*k*_1+_ (× 10^6^ M^−1^ s^−1^)	*k*_1−_ (s^−1^)	*k*_2+_ (s^−1^)	*k*_2+_ (s^−1^)
Best Fit	6.784	53.41	6.802	9.88
% range	0.30	1.00	1.20	0.59

## Discussion

The nature of IDPs is the unstructured functional native state. As compared to globule folded protein, the diffusion search radius for IDPs is considerably increased [31]. Besides, the protein sequences of IDPs are also different from folded proteins in the sense that charged amino acids are significantly populated. The greater capture radius and long-range directional forces lead to the increased association rate constant of IDPs with their partners relative to folded proteins, the so called “fly-casting” mechanism [54]. On the other hand, the flexibility of IDPs actually breaks the boundary of the folded or disordered protein state. The degree of conformational disorder forms the foundation of the continuum model of protein structure [55]. The ensemble structure of IDPs is completely determined by its sequences properties and can be summarized in the diagram-of-states [56] which is the generalization of the original charge-hydropathy plot [57]. At this point, it is of vital importance to relate the ensemble structure of IDPs with their recognition mechanisms and hence with their specific bio-functions with both experimental and simulation efforts [58–60].

In this study, for the thermodynamic and kinetic studies of MoRFs of Chz1 to H2A.Z-H2B linked fragment, we provided a quantitative analysis on the binding-folding reaction with variable ionic strength and urea concentration. By the analysis of the free energy and association rate constant dependence on mean activity coefficient of NaCl and the concentration of urea, the binding—folding reaction was found to be governed by electrostatic interactions in the diffusion step of association, while the conformation rearrangement step was dominated by hydrophobic forces. In addition, the initial point of various kinetic experiments with NaCl did not follow the linear free energy relationship. Since the structure of TBZ did not change much with additional NaCl in solution, this result suggested that an initial conformation selection step of TChz was involved in the association reaction before encounter complex formation. Also, the association rate constant dependence on NaCl mean activity coefficient was negative for *k*_1+_ and positive for *k*_1−_. This suggested the role of electrostatic interactions played in driving and steering the encounter complex state. By extrapolating the association rate constant *k*_1+_ dependence line to physiological condition, it was obvious that the formation of encounter complex was slowed down with the discontinuity of the dependence relationship. Besides, by extrapolating the NaCl mean activity coefficient dependence line of association constant *K*_*A*_ toward physiological condition, the stability of real protein association was decreased as compared with the intercept value. The stability change indicated the stability of protein association was mostly modulated by electrostatic interactions rather than hydrophobic forces in physiological condition. This result was in consistent with NMR results [40]. Moreover, the rate constant dependence of *k*_1+_ and *k*_1−_ on ionic strength was dramatically larger than urea concentration. The more sensitive of rate constants change to ionic strength mean the associated transient complex was enhanced by electrostatic interactions rather than hydrophobic forces. These results were in consistent with the simulation results by considering an initial conformation selection step of Chz1. However, our experimental study invalided the simulation results on the position of the transition state: the rate-limiting step was the second step of conformation rearrangement that governed mainly by hydrophobic forces rather than the first association step dominated by electrostatic potentials.

The binding mechanism in this system was found to be involved with both the conformation selection and the induced-fit models. The first step of association was believed to be a conformation selection step where H2A.Z-H2B selected a specific conformation to evolve to encounter complex. Our previous simulation results had revealed a collapsed structure that was driven by the intra-chain electrostatic forces [34]. Based on this result, we had reasons to believe the first association may be the expansion of this tertiary collapsed structure. With the increasing of ionic strength in solvent the collapsed structure was diminished, therefore the association rate constant dependence on ionic strength was linearized as the experimental results indicated. The last step was the folding and conformation rearrangement of TChz on TBZ which was a hydrophobic driven process.

The kinetic role of electrostatic forces in this system could be divided in to two separate parts, namely, the intra-chain part and the inter-chain part. The intra-chain electrostatic interactions drove the extended ensemble structure of Chz1 to collapse in physiological condition. This collapsed structure was a direct result of the bipolar distribution of Chz1’s charged amino acids. The concept of functional misfolding was developed to describe the formation of nonnative intra-molecular interactions to prohibit highly promiscuous IDPs from unfavorable binding [61]. In this case here, Chz1 might potentially inhibit nonspecific interactions through shielding its own charged amino acids with the formation of the tertiary collapsed structure to avoid unwanted nonspecific electrostatic interactions with DNA or other cellular proteins. The difference in this case lay in that the structure collapse was driven by intra-chain electrostatic forces rather than hydrophobic forces. The specificity difference of Chz1 and Nap1 might be an evidence for this hypothesis. It had been shown that Nap1 could have non-specific interactions with DNA whereas Chz1 did not interact with DNA [21]. This might indicate the functional relevance of the tertiary collapsed native structure ensembles. However, the formation of collapsed structure did slow down the formation of the encounter complex, and further decrease the total association rate. On the other hand, the kinetic role of the inter-chain of electrostatic forces were mainly facilitating the recognition of Chz1 to H2A.Z-H2B and speeding the association. The formation of encounter complex was driven and stabilized by the directional electrostatic forces as seen from the stopped-flow kinetic results. Besides, the final associated complex was also stabilized by the inter-chain electrostatic forces [34, 40]. The contra balance of intra-chain and inter-chain electrostatic forces of Chz1 would possibly ensures the chaperones associated with H2A.Z-H2B with fast speed without loss of specificity.

## Conclusions

In summary, we characterized the binding-folding mechanism of Chz1 binding to H2A.Z-H2B by equilibrium spectra analysis and global numerical fitting to kinetic stopped-flow data at various ionic strengths and denaturant concentrations. A hidden kinetic intermediate was revealed, and was considered to be the encounter complex of Chz1 binding to H2A.Z-H2B. The free energy dependence analysis based on an extended Debye-Hückel model exhibited an initial conformation selection of Chz1 was likely exist before the formation of the intermediate state. Therefore, a mixed mechanism of three steps including both the conformation selection and induced fit was proposed for binding of Chz1 to H2A.Z-H2B. Our association rate constants dependence analysis indicated the encounter complex was driven and steered by electrostatic interactions which was also the main forces that stabilized the associated complex. The findings here demonstrated how the intrinsically disordered protein with bipolar charged amino acids distribution achieved the balance of specificity and fast association through the modulation of intra-chain and inter-chain electrostatic interactions. The native structure ensembles formed collapsed conformation that prohibited non-specific interactions with DNA or other cellular proteins, while the electrostatic enhanced association rate ensured fast binding. Our study also provided insights for *in vivo* situation. Although there were thousands of protein types contained in a single cell, the high affinity between Chz1 and H2A.Z-H2B (∼ 10^6^ M^−1^) and the intrinsically disordered nature of Chz1 ensures high specificity [62]. Therefore, almost all of the histone chaperones Chz1 were associated with H2A.Z-H2B whenever possible in physiological conditions. Given the influence of crowding effects caused by high condensation of cell components, we can still expect the same recognition mechanism *in vivo*, but more data were needed to illustrate this point.

## Supporting information

S1 Fig Equilibrium spectra analysis of TChz binding to TBZ_Y139W_. The NaCl or urea induced CD and fluorescence signal change were plotted together with the best fitting to a two-state equilibrium model. In (A) and (C), fluorescence maximum peak positions and intensities were shown in void dots (fitted with dashed lines) and solid dots (fitted with solid lines), respectively. In (B) and (D), CD molar ellipticity was plotted with solid dots and the fitting lines were shown with solid lines. In all four pictures, TBZ_Y139W_ was represented in navy and TChz-TBZ_Y139W_ complex was represented in dark yellow. Data were recorded in 0.01M PBS (pH 7.4), at 298K.
(EPS)

S2 Fig Spectra overlap of the emission of TBZ_Y139W_ and the UV absorbance of TBZ_*WT*_ (A) and TChz (B). Fluorescence of TBZ_Y139W_ was by excitation at 290nm, and emission spectra was recorded from 305nm to 450nm. The emission spectra of TBZ_Y139W_ was represented with blue line and absorption spectra of TBZ_*WT*_ and TChz were represented with *red* and *green* line, respectively.
(EPS)

S1 File Supporting information file. This file includes the methods used for protein expression and purification, fitting equations for equilibrium two-state dissociation and for fluorescence titration experiments, as well as the Equilibrium spectra analysis of TChz/TBZ_Y139W_ complex association.
(PDF)
