# Supplementary material for: Electrostatic forces govern the binding mechanism of intrinsically disordered histone chaperones
Source: PLoS One. 2017 May 26;12(5):e0178405. doi: 10.1371/journal.pone.0178405 (PMC5446181; doi:10.1371/journal.pone.0178405)
Supplement: S1 File — This file includes the methods used for protein expression and purification, fitting equations for equilibrium two-state dissociation and for fluorescence titration experiments, as well as the Equilibrium spectra analysis of TChz/TBZY139W complex association. (PDF) [file pone.0178405.s003.pdf]

Electrostatic forces govern the binding mechanism of intrinsically disordered histone chaperones

Chuanbo Liu,[†,‡] Tianshu Wang,[‡,¶] Yawen Bai,[§] and Jin Wang[*,‡,¶,‖]

†*University of Chinese Academy of Sciences, Beijing, P.R. China, 130022.*

‡*State Key Laboratory of Electroanalytical Chemistry, Changchun Institute of Applied Chemistry, Chinese Academy of Sciences, Changchun, Jilin, P.R. China, 130022.*

¶*College of Physics, Jilin University, Chuangchun, Jilin, P. R. China, 130012.*

§*Laboratory of Biochemistry and Molecular Biology, Center for Cancer Research, National Cancer Institute, National Institutes of Health, Bethesda, Maryland, USA, 20892.*

‖*Department of Chemistry and Physics, State University of New York, Stony Brook, New York, USA, 11794-3400*

E-mail: Jin.wang.1@stonybrook.edu.

Supporting Information

Protein expression and purification The sequence of the molecular recognition elements (MoRFs) that formed a folded core including residues 71-132 of Chz1 (termed TChz), residues 37-131 of H2B and residues 29-125 of H2A.Z (linked together and termed TBZ), and the mutants of TBZ_{Y139W} were used in this study. *Escherichia coli* BL21(DE3) pLysS cells (Invitrogen) were grown in LB and 2 × TY medium at 37 °C for TBZ (both WT and mutants) and TChz, respectively. Overexpression of the fusion proteins were induced with

1mMisopropyl-β-D-thiogalactopyranoside when A_{600} reached 0.8 - 1.0 at 35 °C overnight. Harvested cells were lysed by sonication in binding buffer (50 mM Tris.Cl pH = 8.0, 300 mM NaCl, 20mM IMDZ, 8M Urea for TChz; and 10 mM Tris.Cl pH = 8.0, 500 mM NaCl, 1mM EDTA for TBZ) followed by centrifugation at 4 °C to remove the precipitation. The supernatant was passed through a 0.22 μM filter before applied to a binding buffer subjected to pre-equilibrated Ni-NTA column (QIAGEN) for TChz or a SP Sepharose High Performance (GE Healthcare) for TBZ, respectively. The elution buffer for TChz contained extra 250 mM imidazole, while for TBZ 1M NaCl was used for a gradient elution. Elution samples were first characterized by SDS-PAGE and then were concentrated and subjected to a Superdex-75 gel-filtration column (GE Healthcare) pre-equilibrated with 0.01 M PBS (pH 7.4). The identity of purified proteins were verified by MALDI-TOF mass spectrometry and sample solutions were concentrated and stored at -80 ° before further usage. The concentrations of all protein samples were determined by BCA assay with a microplate reader (Infinite-200 PRO, TECAN) with BSA as the standard control.

Fitting equations for equilibrium two-state dissociation For reversible two-state denaturation systems, we could write the reaction equations as:

$$[\mathrm{D}] \underset{\mathrm{k_{off}}}{\overset{\mathrm{k_{on}}}{\rightleftharpoons}} [\mathrm{N}]$$

The equilibrium constant was, according to the Boltzmann equation,

$$K_{eq} = \frac{[D]}{[N]} = exp\left(-\frac{\Delta G_{D-N}}{RT}\right)$$

The recorded signal was:

$$Signal = f_N \cdot s_N + f_D \cdot s_D$$

where f_N and f_D represented the fraction weight of the signal and S_N and S_D represented the signal. At the equilibrium condition,

$$
\begin{aligned}
f_D =& \frac{D}{D+N} = \frac{K_{eq}}{K_{eq}+1} \\
f_N =& \frac{N}{D+N} = \frac{1}{K_{eq}+1} \\
s_D =& \alpha_D + \beta_D \cdot P \\
s_N =& \alpha_N + \beta_N \cdot P
\end{aligned}
$$

Therefore,

$$
\begin{aligned}
Signal =& \frac{s_N + s_D \cdot K_{eq}}{1+K_{eq}} \\
=& \frac{(\alpha_N + \beta_N \cdot P) + (\alpha_D + \beta_D \cdot P) \cdot exp\left(-\frac{\Delta G}{RT}\right)}{1 + exp\left(-\frac{\Delta G}{RT}\right)}
\end{aligned}
$$

For urea,

$$\Delta G_{ND}(urea) = m_{ND}^{urea}([Urea]_{1/2} - [Urea])$$

While for salt, assuming the transient complex configuration is identical to the native state, we got,

$$\Delta G_{ND}(salt) = m_{ND}^{salt}\left(ln\gamma_{\pm,1/2}^{el} - ln\gamma_{\pm}^{el}\right)$$

where

$$\gamma_{\pm}^{el} = \frac{A \mid Z_1 Z_2 \mid \sqrt{I}}{1 + Ba\sqrt{I}}$$

Then we got the general formula:

$$Signal = \frac{(\alpha_N + \beta_N \cdot P) + (\alpha_D + \beta_D \cdot P) \cdot exp\left(\frac{m}{RT} \cdot x - \frac{m \cdot x_{1/2}}{RT}\right)}{1 + exp\left(\frac{m}{RT} \cdot x - \frac{m \cdot x_{1/2}}{RT}\right)}$$

Where $m = K \cdot RT$, $[Urea]_{1/2} = E/K$ and x represent $[Urea]$ for urea concentration and $ln\gamma_{\pm}^{el}$ for salt mean activity coefficient, respectively. Thus the fitting equation was chosen to

be:

$$Signal = \frac{(\alpha_N + \beta_N \cdot x) + (\alpha_D + \beta_D \cdot x) \cdot exp\,(K \cdot x - E)}{1 + exp\,(K \cdot x - E)}$$

Fitting equations for fluorescence titration experiments Suppose the original concentration of A was A_0, of B was B_0, then the concentration of A after n drops of B was:

$$[A] = \frac{A_0 V_A}{V_A + nV_B}$$

The concentration of B after n drops of B was:

$$[B] = \frac{B_0 n V_B}{V_A + nV_B}$$

By setting $[B]/[A] = x$, we got,

$$n = \frac{A_0 V_A x}{B_0 V_B}$$

Thus,

$$[A] = \frac{A_0}{1 + A_0 x / B_0}$$
$$[B] = \frac{A_0 x}{1 + A_0 x / B_0}$$

From an equilibrium experiment:

$$([A] - c)([B] - c) = k_d c$$
$$c = (A + B + k_d)/2 - \left[(A + B + k_d)^2/4 - (AB)\right]^{1/2}$$

Therefore the fitting equations for equilibrium titration experiments was chosen to be:

$$F = \left\{\rho/2 - \left[\rho^2/4 - \sigma \cdot x\right]^{1/2}\right\} \cdot \Delta F + F_0$$

where,$\rho = (1 + x) \cdot \sigma + k_d$, $\sigma = \frac{A_0 B_0}{B_0 + A_0 x}$.

Equilibrium spectra analysis of TChz/$\mathbf{TBZ_{Y139W}}$ complex association By detailed analysis of the fluorescence and CD spectra, some vital information could be deduced for this histone chaperone interaction system. First of all, fluorescence spectra at the physiological experimental conditions were shown in S1 Fig (A) and S1 Fig (C) that the binding of TChz significantly increased the emission fluorescence intensity of $\mathrm{TBZ_{Y139W}}$. Secondly, as for histone heterodimer fragment $\mathrm{TBZ_{Y139W}}$, the NaCl induced fluorescence intensity and maximum peak wavelength (λ_{max}) shift exhibited a down-hill like behavior accompanied with similar CD decrease, suggesting the secondary structure increase of $\mathrm{TBZ_{Y139W}}$ was correlated with fluorescence signal decrease. Moreover, urea induced denaturation of the $\mathrm{TBZ_{Y139W}}$ demonstrated a large λ_{max} red shift, and increased fluorescence intensity along with the protein structure transformation to a less compact structure state. Finally, λ_{max} of $\mathrm{TBZ_{Y139W}}$ and TChz-$\mathrm{TBZ_{Y139W}}$ had almost the same value within the experimental error (± 1nm), indicating an excited-state formation complex was not likely. Since fluorescence signal was highly sensitive to the micro-environment around the fluorephore, especially for Trp139 in this case, the peak position shift of Trp containing protein could only be interpreted by the change of the surrounding hydrophobicity. Therefore a red shift of λ_{max} means that Trp139 was placed in a more hydrophilic environment, i.e. having more contacts with the solvent molecules hence influenced by larger solvent relaxation effect. Thus we had reasons to believe the energy of excited Trp139 of $\mathrm{TBZ_{Y139W}}$ was transferred to its own adjacent radiationless acceptors, while with the binding of TChz this energy transfer was interfered. As a matter of fact, partial overlap of spectra were observed for both TChz and TBZ_{WT} with $\mathrm{TBZ_{Y139W}}$ S2 Fig, indicating energy transfer was possible for TChz-Trp139 and TBZ_{WT}-Trp139. Then the increasing of fluorescence intensity due to TChz binding could be interpreted as that the binding of TChz reduced this resonance energy transfer of $\mathrm{TBZ_{Y139W}}$ by meddling with its local structure around Trp139.

By increasing NaCl concentration from physiological condition to extra 1M high salt solution environment, the fluorescence intensity of TBZ_{Y139W} was decreased to 78.8% of initial intensity along with a blue shift of the maximum fluorescence emission peak wavelength. Furthermore, the CD signal was also significantly reduced which suggested that TBZ_{Y139W} was getting more compact with increasing NaCl concentration. These evidences indicated that the fluorescent amino acid Trp139 was buried inside of the protein hydration shell due to the salt induced structure contraction. However, with the increasing of NaCl concentration, λ_{max} of TChz-TBZ_{Y139W} complex appeared to be a reciprocate type movement by first showing a small red shift from 332 nm to 336 nm ($<$ 0.3 M NaCl) and then a large blue shift from 336 nm to 323 nm (0.3 M $\sim$ 1 M). Moreover, the conformation transition pattern was changed from a down-hill like behavior to a two-state equilibrium transition as seen from both the fluorescence and CD spectra. Since the experiments were performed in the same solution conditions, the only reason for this spectroscopic difference was the presence of TChz. As a matter of fact, the increasing of NaCl concentration in solvent influenced both the intramolecular and the intermolecular interactions within TBZ_{Y139W} and with TChz, respectively. But for the specific point of Trp139, when NaCl concentration was less than 0.3 M, the red shift of λ_{max} clearly indicates that Trp139 was exposed to a more hydrophilic micro-environment which could only be interpreted by the dissociation of the middle motif of TChz. Furthermore, as the CD signal was decreased after that NaCl concentration was increased larger than 0.3 M with the TBZ_{Y139W} CD signal merely changed, we can conclude TChz was not completely dissociated from TBZ_{Y139W} at this point. This conformation was distinctly different from the native state of TChz-TBZ_{Y139W} complex in physiological condition, leading to an intermediate that had not been reported. However, due to the limitation of ensemble spectroscopic techniques, whether N-terminal or C-terminal of TChz was dissociated from TBZ_{Y139W} or both still were in contact with TBZ_{Y139W} could not be deduced from the current experiment data. This would be further illustrated by our next experimental project. When NaCl concentration was increased to larger than 0.3 M, the fluorescence and

CD signal behavior of TChz-TBZ_{Y139W} complex became very similar to TBZ_{Y139W}. Note that the fluorescence intensity of TChz-TBZ_{Y139W} complex was smaller than TBZ_{Y139W} even at 1 M NaCl concentration, suggesting the presence of TChz still had quenching effect on Trp139 at this high salt solvent condition. Using the same argument, we can conclude that when urea concentration < 3 M, TChz was not completely detached from TBZ_{Y139W} and also intramolecular resonance energy transfer of Trp139 was enhanced with the presence of TChz, probably due to the structural stabilization of the intermolecular electrostatic forces. Similar to NaCl induced fluorescence signal change, when urea concentration was larger than 3 M, TChz still had nonspecific interactions with TBZ_{Y139W} and acted like a quencher to Trp139.
